# Specific Autoantibodies and Clinical Phenotypes Correlate with the Aberrant Expression of Immune-Related MicroRNAs in Dermatomyositis

**DOI:** 10.1155/2019/2927061

**Published:** 2019-02-19

**Authors:** Lifang Ye, Yu Zuo, Hanbo Yang, Wenli Li, Qinglin Peng, Xin Lu, Guochun Wang, Xiaoming Shu

**Affiliations:** ^1^Department of Rheumatology, Beijing Key Lab for Immune-Mediated Inflammatory Diseases, China-Japan Friendship Hospital, Yinghua East Road, Chaoyang District, Beijing 100029, China; ^2^Peking University China-Japan Friendship School of Clinical Medicine, Beijing 100029, China

## Abstract

**Aims:**

The serum concentrations of miRNAs, miR-23a-3p, miR-23b-3p, miR-146a-5p, miR-146b-5p, and miR-150-5p, were shown to be associated with the immune and inflammatory progressions. We assessed the expressions of these five miRNAs in association with clinical phenotypes and myositis-specific autoantibody-defined subgroups of dermatomyositis (DM).

**Methods:**

The present study included 49 patients with DM and 30 healthy controls. The serum concentrations of miR-23a-3p, miR-23b-3p, miR-146a-5p, miR-146b-5p, and miR-150-5p were measured by quantitative reverse transcription polymerase chain reaction (qRT-PCR). Associations between the serum concentrations of miRNAs and DM clinical immune phenotypes were examined as well.

**Results:**

The serum concentrations of miR-23b-3p, miR-146a-5p, and miR-150-5p were significantly downregulated in DM patients (*P* < 0.001, *P* < 0.001, and *P* = 0.002, respectively), while miR-146b-5p was remarkably upregulated in DM patients compared with healthy controls (*P* = 0.039). Similarly, the expressions of miR-23b-3p, miR-146a-5p, and miR-150-5p were significantly downregulated in the peripheral blood mononuclear cells (PBMCs) from DM patients. Further study indicated that the serum level of miR-23b-3p was significantly correlated with creatine kinase (CK) (*r* = −0.286, *P* = 0.046) and the serum level of miR-146a-5p was evidently correlated with C-reactive protein (CRP) (*r* = −0.358, *P* = 0.012). Significant correlations were also observed between the serum levels of miR-146b-5p and CRP (*r* = −0.347, *P* = 0.014) and the erythrocyte sedimentation rate (ESR) (*r* = −0.287, *P* = 0.046). In addition, the expression level of miR-146b-5p was upregulated in DM complicated by tumors compared with those without tumors (*P* = 0.001 and *P* < 0.001, respectively). Especially, miR-150-5p was significantly downregulated in DM patients with anti-MDA5 antibodies and anti-NXP2 antibodies compared with those without (*P* = 0.017 and *P* = 0.047, respectively). No significant differences were observed between the four serum microRNAs in patients with and without interstitial lung diseases (all *P* > 0.05).

**Conclusion:**

The results suggest an association between the four immune-related microRNAs and different clinical immune-phenotypes, and this association may regulate the complexity of disease processes through multipathways in DM patients.

## 1. Introduction

Dermatomyositis is a heterogeneous group of autoimmune inflammatory disorders with a broad range of symptoms, variant organ involvement, disease severities, and outcome, which can be subclassified on the basis of clinical manifestations and myositis-specific autoantibodies (MSAs) [1]. Although researchers thought that its heterogeneity might be characterized by specific genetic factors involved in the different regulated networks, the basis for the distinctive MSA profile and its regulation in DM patients is poorly understood [2].

MicroRNAs (miRNAs) are the key regulators for the expression of related target genes, and the aberrant expression in the immune system may be associated with several human diseases, including inflammation, interstitial lung disease, and autoimmune diseases [3–6]. In the past ten years, identification of differentially expressed microRNAs (miRNAs) in muscle biopsy samples from patients with inflammatory myopathies caused those miRNAs to be considered new potential molecular pathogenesis or prognostic biomarkers for disease development and progression. Eisenberg et al. firstly reported that several microRNAs were up- and downregulated in the muscle tissues of polymyositis and dermatomyositis (PM/DM) [7]. In addition, miR-146a and miR-146b were found to be upregulated in the muscle tissues in polymyositis/dermatomyositis (PM/DM). However, miR-146a was downregulated in the study conducted by Yin et al. from China [8]. To date, idiopathic inflammatory myopathy- (IIM-) related miRNAs were found by different levels of expressions in the whole blood, peripheral blood mononuclear cells, skeletal muscles, plasma, and serum [9, 10]. Misunova et al. identified serum Let-7b and miR-3907 upregulated and miR-4299 downregulated in DM patients and miR-3907 associated with disease activity [11]. However, these studies did not take into account the diverse myositis-specific autoantibodies which were associated with a distinctive pattern of disease or phenotype [12]. Certain autoantibodies from patients with rheumatic diseases including systemic lupus erythematosus (SLE) have been shown to target key components of microRNA (miRNA) generation [13]. These studies stimulated us to investigate whether different miRNA-mediated regulations exist in DM patients with distinct myositis-specific antibody (MSA) status.

Recently, Prabahar et al. firstly developed an immune-related miRNA database called ImmunemiR. In this database, a total of 245 immune-related miRNAs were recorded within 92 immune-related diseases. Among them, 78% of the immune-related miRNAs were associated with autoimmune diseases which mainly include rheumatoid arthritis (RA), systemic lupus erythematous (SLE), and other connective tissue diseases [3]. However, it may be less significant to measure the level of only one of the 245 immune-related miRNAs. According to the ImmunemiR database, miR-23a-3p, miR-23b-3p, miR-146a-5p, miR-146b-5p, and miR-150-5p were dysregulated in kinds of autoimmune diseases. However, there is limited information on serum miR-23a-3p, miR-23b-3p, miR-146a-5p, miR-146b-5p, and miR-150-5p associated with clinical phenotypes and MSAs in DM. Therefore, in this condition, we focused on the interactions among multiple miRNAs and examined a possibility that the expression pattern of multiple miRNAs in each patient could be utilized as a more reliable clinical phenotype marker for DM.

In this study, we hypothesized that these five immune-related miRNAs, including miR-23a-3p, miR-23b-3p, miR-146a-5p, miR-146b-5p, and miR-150-5p, may be associated with different clinical phenotypes and autoantibody statuses. Our hypothesis is that patients with different characteristics and MSA profiles are characterized by specific immune-related miRNA profiles.

## 2. Material and Methods

### 2.1. Patients

We selected 49 patients with DM who were hospitalized in China-Japan Friendship Hospital from March 2011 to February 2016 and then collected their serum samples. To further qualify the expressions of microRNAs in DM, we also studied the expression in PBMCs from another 23 patients with DM who were hospitalized in China-Japan Friendship Hospital from November 2016 to August 2018 (clinical data were shown in supplementary material (available here)). All patients fulfilled the criteria of definitive diagnosis of DM according to the Bohan and Peter criteria and 2017 European League Against Rheumatism/American College of Rheumatology Classification Criteria for Adult and Juvenile Idiopathic Inflammatory Myopathies and Their Major Subgroups. All patients were not complicated by infections and other connective tissue diseases. A summary of the clinical and laboratory features of DM patients enrolled in this study is shown in Table 1. This study also included 39 healthy controls, who were gender-matched with the patients. Among them, sera from 30 healthy controls and PBMCs from 9 healthy controls were analyzed. Written informed consent was obtained from all participants, and this study was approved by the Ethics Committee of China-Japan Friendship Hospital (Beijing, China).

### 2.2. Clinical Assessment

Completing medical histories, physical examinations, and laboratory tests was conducted to all patients during the first visit. Routine laboratory assessments were performed, including the determination of the levels of serum creatine kinase (CK), aspartate aminotransferase (AST), lactate dehydrogenase (LDH), erythrocyte sedimentation rate (ESR), C-reactive protein (CRP), ferritin, high-resolution computerized tomography (HRCT), pulmonary function tests (PFT), and muscle biopsies at the time of visiting at China-Japan Friendship Hospital. ILD was diagnosed based on the findings of HRCT of the chest and pulmonary function tests. ILD classification is based on the clinical-imaging-pathological diagnostic criteria revised by the American Thoracic Society (ATS) and the European Respiratory Society (ERS) in 2013 [14]. All malignancies were confirmed by histopathological findings. The disease activity of patients with DM was evaluated using a visual analogue scale (Physician's Global Assessment (PGA)) (range: 0–10 cm).

Serum MSAs including anti-Mi-2*α*, anti-Mi-2*β*, anti-TIF1-*γ,* anti-NXP2, anti-SAE1, anti-MDA5, anti-SRP, anti-Jo-1, anti-PL-7, anti-PL-12, anti-OJ, and anti-EJ were detected using commercially available kits (EUROIMMUN, Lübeck, Germany). Anti-HMGCR autoantibodies were detected by the ELISA kit (enzyme-linked immunosorbent assay) (Inova Diagnostics Inc., San Diego, CA, USA).

### 2.3. Isolation of Serum RNA and PCR

Total RNA was extracted from 1 mL serum using a TRIzol reagent (Invitrogen Corp., Carlsbad, CA, USA) according to the manufacturer's instructions and eventually was dissolved in 20 *μ*L RNase-free water. The concentration and purity of the small RNAs were measured with an ultraviolet spectrophotometer (Kaiao, Beijing, China). The ratio of 260/280 was 1.2–2.1. Reverse transcription was performed using the TIANGEN Reverse Transcription kit (TIANGEN Biotech Co. Ltd., Beijing, China) according to the manufacturer's instructions. The iQTM SYBR Green Supermix (Bio-Rad Laboratories Inc., Hercules, CA, USA) was used in the quantitative reverse transcription-polymerase chain reaction (qRT-PCR) for relative quantification of miR-23a-3p, miR-23b-3p, miR-146a-5p, miR-146b-5p, and miR-150-5p in our study with cel-miR-39-3p as an internal control. The primer sequences were specifically designed by RiboBio company (Guangzhou RiboBio Co. Ltd.), which include Bulge-Loop TM hsa-miR-23a-3p RT Primer (ssD809230260), Bulge-Loop TM hsa-miR-23a-3p Forward Primer (ssD809230952), Bulge-Loop TM hsa-miR-23b-3p RT Primer (ssD809230261), Bulge-Loop TM hsa-miR-23a-3p Forward Primer (ssD809230953), Bulge-Loop TM hsa-miR-146a-5p RT Primer (ssD809230159), Bulge-Loop TM hsa-miR-146a-5p Forward Primer (ssD809230851), Bulge-Loop TM hsa-miR-146b-5p RT Primer (ssD809230161), Bulge-Loop TM hsa-miR-146b-5p Forward Primer (ssD809230853), Bulge-Loop TM hsa-miR-150-5p RT Primer (ssD809230169), Bulge-Loop TM hsa-miR-150-5p RT Primer (ssD809230861), and Bulge-Loop TM miR-Reverse Primer (ssD089261711).

The 10 *μ*L of reaction solutions included 1 *μ*L diluted cDNA templates, 0.2 *μ*L reverse primer, 0.2 *μ*L sense primer, 5 *μ*L of SYBR Green Supermix, and a final volume with RNase-free water. qRT-PCR amplification was performed by using the CFX96 RT-PCR System (Bio-Rad Laboratories Inc., Hercules, CA, USA) according to the manufacturer's instructions. The reactions were incubated in an optical 96-well reaction plate at 95°C for 5 min, followed by 40 cycles at 95°C for 20 s, at 60°C for 20 s, and at 70°C for 10 s. No signal was detected in negative control (no reverse transcriptase or no template). PCR was simultaneously performed in triplicate for each sample for both the miRNAs and cel-miR-39-3p. To validate the accuracy and specificity of the expected PCR product, we performed the melting curve analysis. Relative miRNA production was determined with the aid of the 2^-ΔΔCt^ method, where Ct was the threshold cycle. Differences in miR-23a-3p, miR-23b-3p, miR-146a-5p, miR-146b-5p, and miR-150-5p contents in patients with DM compared with the controls were expressed as fold changes.

### 2.4. Isolation of Human PBMCs and miRNA qRT-PCR

PBMCs were obtained from EDTA-anticoagulated blood by Isopaque-Ficoll gradient centrifugation (Sigma-Aldrich). Cells were cryopreserved at -80°C for quantitative real-time PCR (qRT-PCR) analysis. The total PBMC RNAs were extracted by the miRNeasy kit (QIAGEN). To evaluate the miRNA expression levels, qRT-PCR was implemented with Mir-X™ miRNA First-Strand Synthesis Kit and Mir-X™ miRNA qRT-PCR TB Green™ Kit (Takata, Japan). The cycling condition was as follows: 95°C for 30 sec, 95°C for 5 sec (40 cycles), and 60°C for 34 sec. qRT-PCR amplification was performed by using the ABI7500 RT-PCR System (Applied Biosystems, USA) according to the manufacturer's instructions. The expression of the gene was standardized to the expression amount of U6 and analyzed through the 2^-ΔΔCt^ method. The primer sequences (from 5′ to 3′) are listed in Table 2.

### 2.5. Statistical Analysis

Continuous variables are presented as mean ± standard deviation (SD) or medians and interquartile range (IQR) and frequency and proportions for categorical variables. Categorical data were analyzed using Fisher's exact test. Comparisons of continuous data were made using the Mann-Whitney *U* test for two independent samples of data. Multiple comparisons for continuous variables were performed using the analysis of variance (ANOVA). Additionally, correlations with clinical data were assessed using Spearman's rank correlation coefficient. Statistical analyses were performed using the SPSS 19.0 software (SPSS Inc., Chicago, IL, USA). GraphPad Prism 5 (version 5.01) was used for plotting data. Two-tailed *P* values less than 0.05 were statistically considered significant.

## 3. Results

### 3.1. Clinical Features of the DM Patients

Characteristics of the DM patients are shown in Table 1. Here, 49 DM patients including 25 patients with ILD and 24 patients without ILD were enrolled. The mean age of all patients at the disease onset was 47.26 ± 11.44 years. The median duration of the DM disease was 19.04 months. DM with ILD and DM without ILD subgroups were of similar age, race, and sex. The level of ESR in the DM with ILD group was significantly higher than that in DM without ILD (*P* < 0.01). Although the CK levels in DM with ILD were lower than those in DM without ILD, there was no significant difference between both of them (*P* > 0.05). The serum ferritins were 612.9 ± 624.3 ng/mL in DM with ILD, which were higher than that in patients without ILD. The prevalence of MSAs in the enrolled patients was 81.6%. The rates of the myositis-specific autoantibodies in DM patients are summarized in Table 1. Intriguingly, a patient who was diagnosed with DM without ILD has been found to have anti-MDA5 antibody-positive DM. An anti-TIF1-*γ* antibody, anti-NXP2 antibody, and HMGCR antibody were mainly found in the DM without ILD groups. There was no statistical difference in the physician's global assessment and the visual analogue scale (PGA-VAS) between DM with ILD and DM without ILD.

### 3.2. Different Expression Levels of Immune-Related miRNAs in DM Patients

**Figure 1:**
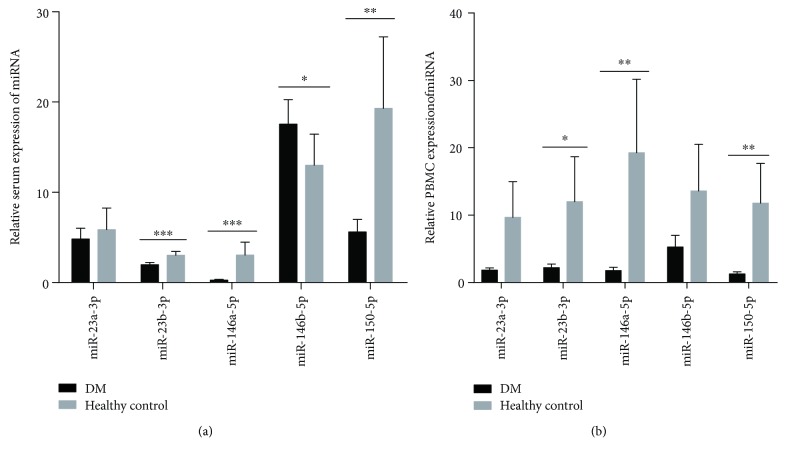
Comparison of the levels of microRNAs (miR-23a-3p, miR-23b-3p, miR-146a-5p, miR-146b-5p, and miR-150-5p) in sera of DM patients and the healthy controls (49 DM and 30 control cases) and in PBMCs between 23 DM patients and 9 healthy controls by quantitative reverse transcription-PCR (qRT-PCR). Values shown were normalized to cel-miR-39-3p. Differences between the levels were expressed as relative expression using the 2^-ΔΔCt^ method. Data were expressed as mean ± standard deviation (SD). 2-group comparisons were analyzed using the Mann-Whitney *U* test. All *P* values were two-sided, and *P* < 0.05 was statistically considered significant. ^∗∗∗^ *P* < 0.001, ^∗∗^ *P* < 0.01, and ^∗^ *P* < 0.05. miR: microRNA.

Five immune-related miRNAs including miR-23a-3p, miR-23b-3p, miR-146a-5p, miR-146b-5p, and miR-150-5p were analyzed by qPCR in the sera of 49 patients with DM and compared with those of 30 healthy controls (Figure 1(a)). The expression levels of miR-23b-3p, miR-146a-5p, and miR-150-5p were significantly downregulated in the sera of DM (1.99 ± 0.87, 0.26 ± 0.29, and 5.61 ± 4.85, respectively) compared with the HCs (3.02 ± 1.13, 3.04 ± 3.82, and 19.3 ± 21.2, respectively) (*P* < 0.001, *P* < 0.001, and *P* = 0.002, respectively). However, the serum levels of miR-146b-5p in patients with DM were significantly upregulated compared with HCs (17.54 ± 9.44 and 12.99 ± 9.20, respectively, *P* = 0.039). There was no statistical difference between the naive-treatment patients and treated patients in these miRNAs (data not shown).

Additionally, we detected the expression levels of these five microRNAs in PBMCs from another 23 patients and 9 healthy controls (Figure 1(b)). Similarly, the expression levels of miR-23b-3p, miR-146a-5p, and miR-150-5p were significantly downregulated in the PBMCs of DM (2.20 ± 0.52, 1.76 ± 0.48, and 1.27 ± 0.30, respectively) compared with the HCs (12.00 ± 6.64, 19.26 ± 10.91, and 11.76 ± 5.89, respectively) (*P* = 0.038, *P* = 0.002, and *P* = 0.002, respectively). But miR-23a-3p and miR-146b-5p were not found to be statistically different between the patients and the healthy controls.

### 3.3. Correlations between the Levels of MicroRNAs (miR-23b-3p, miR-146a-5p, miR-146b-5p, and miR-150-5p) and the Clinical Laboratory Parameters of DM

**Figure 2:**
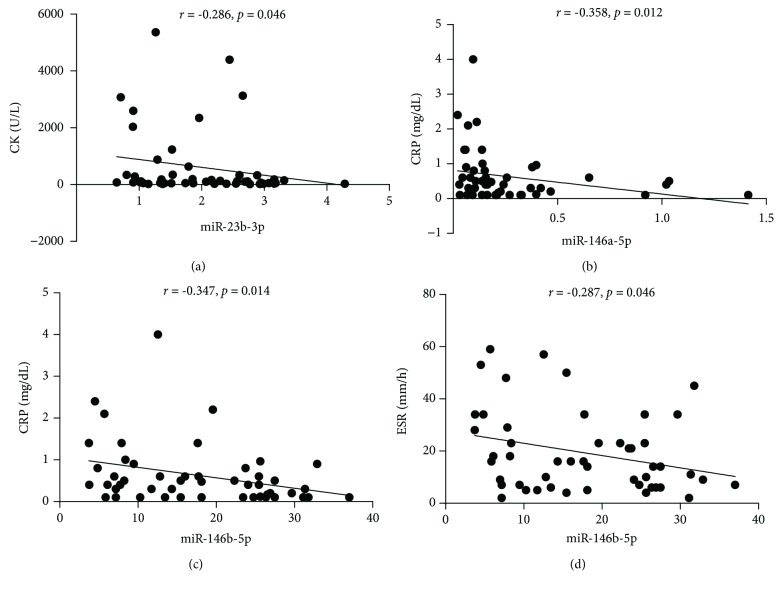
Correlations between miR-23b-3p, miR-146a-5p, and miR-146b-5p and clinical laboratory parameters of DM. (a) The results showed that miR-23b-3p expressions were negatively correlated with CK (*r* = −0.286, *P* = 0.046). (b) Serum miR-146a-5p levels were significantly correlated with CRP (*r* = −0.358, *P* = 0.012). (c, d) Significant correlations were observed between miR-146b-5p levels and CRP (*r* = −0.347, *P* = 0.014) and ESR (*r* = −0.287, *P* = 0.046). Data were analyzed by Spearman's rank correlation coefficient. All *P* values were two-sided, and *P* value < 0.05 was statistically considered significant. DM: dermatomyositis; CRP: C-reactive protein; ESR: erythrocyte sedimentation rate; CK: creatine kinase; LDH: lactate dehydrogenase.

To study whether there were correlations between the dysregulation of the immune-related miRNAs and the different clinical laboratory parameters of DM, correlation analyses between the miRNA levels and CK, lactate dehydrogenase (LDH), ESR, CRP, and ferritins were tested during their first visit. The results showed that the expression levels of serum miR-23b-3p were negatively correlated with CK (*r* = −0.286, *P* = 0.046, Figure 2(a)), while there were no significant correlations with the ESR, CRP, and LDH. Serum miR-146a-5p levels were rather negatively related to CRP (*r* = −0.358, *P* = 0.012, Figure 2(b)), while they were not correlated with ESR, CK, and LDH. Significant correlations were found between the miR-146b-5p levels and the CRP (*r* = −0.347, *P* = 0.014, Figure 2(c)) and ESR (*r* = −0.287, *P* = 0.046, Figure 2(d)), while there was no significant difference in CK and LDH. Serum levels of miR-150-5p did not reach statistical significance compared to the associated clinical laboratory parameters.

### 3.4. Associations between Four Immune-Related miRNAs and Myositis-Specific Autoantibodies

**Figure 3:**
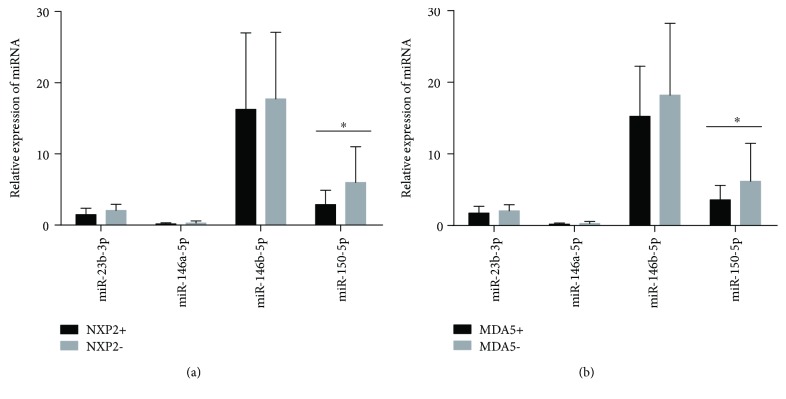
Associations between serum miRNA levels and MSAs. (a) Serum miR-150-5p levels were significantly downregulated in DM patients with anti-NXP2 antibodies (3.58 ± 2.02, 11 cases) compared with those without anti-NXP2 antibodies (6.19 ± 5.27, 38 cases; *P* = 0.047). (b) Serum miR-150-5p levels were also lower in DM patients with anti-MDA5 antibodies (3.58 ± 2.02, 11 cases) than in those without anti-MDA5 antibodies (6.19 ± 5.27, 38 cases; *P* = 0.017). Then, 2-group comparisons were performed using the Mann-Whitney *U* test. All *P* values were two-sided, and *P* value < 0.05 was statistically considered significant. MDA5: melanoma differentiation-associated gene 5; NXP2: nuclear matrix protein-2.

To study whether different immune-related miRNAs could contribute to the MSAs in DM, we explored the four serum levels of immune-related miRNAs in DM patients with different MSA profiles. As shown in Figure 3, serum miR-150-5p levels were significantly downregulated in DM patients with anti-NXP2 antibodies (3.58 ± 2.02, 11 cases) compared with those without anti-NXP2 antibodies (6.19 ± 5.27, 38 cases; *P* = 0.047) (Figure 3(a)). In addition, serum miR-150-5p levels were also significantly downregulated in DM patients with anti-MDA5 antibodies (3.58 ± 2.02, 11 cases) compared with those without anti-MDA5 antibodies (6.19 ± 5.27, 38 cases; *P* = 0.017) (Figure 3(b)). However, no significant differences were observed between the serum levels of miR-23b-3p, miR-146a-5p, and miR-146b-5p in DM patients with MSAs (anti-ARS, anti-SAE, anti-TIF1-*γ*, anti-Mi-2, anti-SRP, and HMGCR autoantibodies) and those patients without MSAs (data not shown). However, we did not find significant correlations between these five immune-related miRNA expressions of PBMCs and MSAs in DM patients (data not shown).

### 3.5. Correlations between the Levels of MicroRNAs (miR-23b-3p, miR-146a-5p, miR-146b-5p, and miR-150-5p) and Organ Involvement in DM Patients

**Figure 4:**
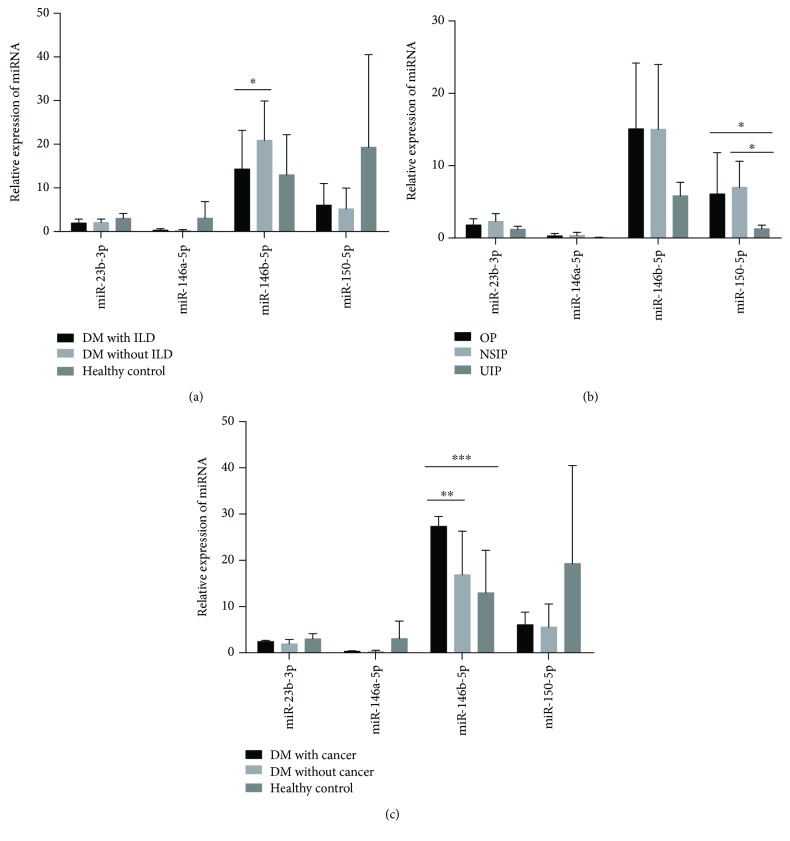
Correlations between serum miRNA levels and organ involvement in DM patients. (a) Significant difference was observed between expression levels of serum miR-146b-5p in DM patients with and without ILD (*P* = 0.013). (b) The serum levels of miR-150-5p were significantly higher in DM patients with NSIP than in those with UIP (*P* = 0.044 and *P* = 0.029, respectively). (c) The expression levels of serum miR-146b-5p were higher in DM patients with cancer than in those without cancer and HCs (*P* = 0.001 and *P* < 0.001, respectively). Multiple comparisons for continuous variables were performed using the analysis of variance (ANOVA). ^∗∗∗^ *P* < 0.001, ^∗∗^ *P* < 0.01, and ^∗^ *P* < 0.05.

We further examined whether organ involvement would be associated with specific miRNAs in DM patients. As shown in Figure 4(a), no significant differences were observed between the serum levels of miR-23b-3p, miR-146a-5p, and miR-150-5p in DM patients with and without ILD. Significant difference was observed only between serum levels of miR-146b-5p in DM patients with and without ILD (*P* = 0.013); however, it did not reach statistical significance between DM patients with ILD and HCs (*P* > 0.05). To verify whether the types of ILD could be associated with specific miRNAs in DM, we further categorized ILD into organized pneumonia (OP, *n* = 15), nonspecific interstitial pneumonia (NSIP, *n* = 8), and usual interstitial pneumonia (UIP, *n* = 2). The serum levels of miR-150-5p were significantly higher in DM with NSIP or OP than those with UIP (*P* = 0.044 and *P* = 0.029, respectively, Figure 4(b)). In addition, no significant differences were observed between the serum levels of miR-23b-3p, miR-146a-5p, and miR-146b-5p in DM patients with NSIP, OP, or UIP.

It has been extensively reported that cancer was a severe complication which increased mortality in DM patients. However, no attempt has been made to evaluate the associations between immune-related miRNAs and cancer in DM patients. Hence, we simultaneously explored the correlations between the candidates of four immune-related miRNAs and cancer in DM patients. As shown in Figure 4(c), the expression levels of serum miR-146b-5p were higher in DM patients with cancer than those without cancer and HCs (*P* = 0.001 and *P* < 0.001, respectively). We did not observe associations between the expression levels of serum miR-23a-3p, miR-146a-5p, and miR-150-5p in DM patients with cancer and those without.

## 4. Discussion

Our study shows that the four immune-related miRNAs are differentially expressed in DM patients. The major findings in this study indicated that (1) decreased expression level of serum miR-23b was negatively correlated with CK levels, (2) decreased expression level of serum miR-150-5p was specifically correlated with anti-MDA5 and anti-NXP2 autoantibodies in DM patients, and (3) expression level of serum miR-146b-5p was significantly higher in DM patients with cancer than those without, while these four immune-related miRNAs were not significantly associated with ILD in DM patients. These results indicated that immune-related miRNAs could reflect the different phenotypes in DM patients.

**Figure 5:**
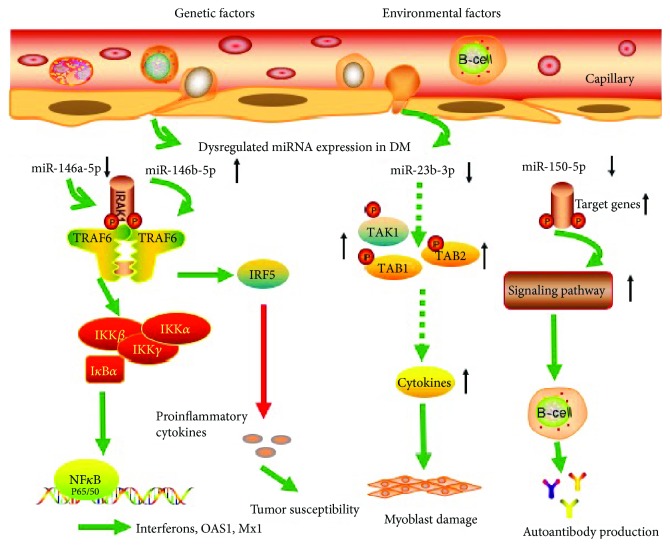
Schematic diagram of the regulation role of four immune-related miRNAs in DM. IRAK1: interleukin-1 receptor-associated kinase 1; TRAF6: tumor necrosis factor receptor-associated factors 6; IKK*α*: inhibitor kappa B kinase *α*; IKK*β*: inhibitor kappa B kinase *β*; IKK*γ*: inhibitor kappa B kinase *γ*; IkB*α*: inhibitor k binding *α*; IRF6: interferon regulatory factor 6; NF-*κ*B: nuclear factor-k-gene binding; TAK1: transforming growth factor-activated kinase-1; TAB1: transforming growth factor-beta-activated protein kinase 1-binding protein 1; TAB2: transforming growth factor-beta-activated protein kinase 1-binding protein 1; TAK1: transforming growth factor-*β*-activated kinase 1.

DM is recognized to be a heterogeneous disease, which is associated with MSAs and possibly consists of distinct disease entities such as antisynthetase syndrome (ASS), anti-MDA5-positive DM, and cancer-associated DM. In agreement with this, our results show that the serum levels of immune-related miRNAs clearly differed between the clinical phenotypes and autoantibody groups. In particular, patients with anti-MDA5 and anti-NXP2 autoantibodies presented downregulated miR-150-5p. So far, however, no data are yet available on the biological effects of microRNAs regarding the autoantibodies in DM. Our study firstly described that MSAs correlated with the immune-related miRNAs in DM. Wuttge et al. reported that circulating miRNAs were different between systemic sclerosis with ACA- and anti-DNA topoisomerase I antibody-positive patients and those without [15]. Our results were indirectly proven by the recent reported observations that the expression of miR-150-5p in the peripheral mononuclear leukocytes (PBMCs) from primary Sjögren's syndrome (pSS) was significantly downregulated compared to the healthy controls, which was potentially linked to B cell functions [16]. Inversely, Chauhan et al. reported that different sets of miRNAs were dysregulated in SLE patients with different autoantibody profiles. One of the miRNAs was miR-150 upregulated in anti-dsDNA+ENA+SLE patients compared with the healthy controls [17]. However, the mechanism by which miRNA participates in the formation of autoantibodies is still not clear. Xia et al. studied that microRNA-326 could upregulate B lymphocyte activity and autoantibody production through regulating Ets-1 in lupus disease [18]. Decreased miR-1246 expression through the AKT-P53 signaling pathway and the downstream effect on the expression of EBF1 leads to further activation of B cells in SLE [19]. Although the exact regulatory mechanism of miRNAs on the autoantibody production was not understood, these data truly suggest that autoimmune rheumatic diseases may be linked at molecular epigenetic levels through their circulating miRNA profiles to autoantibody profiles. Previous studies showed that miR-150 was selectively expressed in mature, resting B and T cells and also affected B cell differentiation and development, which indicated that dysregulation of miR-150 strikingly affected antibody production [20 *–* 22]. These studies provide us solid theories to further study the exact pathogenesis of regulatory B cells and autoantibody production in DM. We speculate that decreased expression of miR-150-5p controls B cell development and autoantibody production through regulating target genes (Figure 5). Further study may be required to explore the exact mechanism of downregulated serum miR-150-5p control MSA especially anti-MDA5 and anti-NXP2 autoantibody production in DM.

There were several studies which investigated the correlations between serum microRNAs and clinical phenotypes in DM patients (Table 3) [8, 11, 23–25]. Inoue and colleagues suggested that the serum and the skin expression of miR-233 were both downregulated in DM patients with Gottron's papules and clinically amyopathic DM patients [24]. Several serum microRNAs, such as miR-7 and miR-21-5p, were observed with dysregulation in DM patients. Upregulation of serum miR-21-5p was correlated with serum immunoglobulin G levels, while a decrease in serum expression of miR-7 was associated with skin involvement in DM patients [25, 26]. These studies, therefore, may show that the serum levels of microRNAs are useful markers for the diagnosis or evaluation of different clinical phenotypes in DM patients.

We firstly found that serum miR-23b-3p levels were correlated significantly with CK, but its role in the skeletal muscle damage in DM is still unknown. There is an interesting study that shows urine miR-23b-3p was significantly downregulated in nonambulant Duchenne muscular dystrophy (DMD) patients compared with age-matched healthy controls [27]. This study suggested that the downregulation of miR-23b-3p was a marker of skeletal damage, which is similar to our study. In addition, several studies have also revealed that miR-23b-3p may exert an effect on different types of immune cells, including monocytes, macrophages, and dendritic cells (DCs) [28, 29]. Moreover, it has been reported that miR-23b-3p prevents multiple autoimmune diseases through the regulation of inflammatory cytokine pathways, such as NF-*κ*B, tumor necrosis factor- (TNF-) *α*, interleukin- (IL-) 1*β*, and IL-17 [30]. Innate immune cells such as DCs and macrophages and inflammatory mediators such IL-17 and TNF-*α* have been shown to be involved in the pathogenesis of skeletal muscle damage, which leads to high serum CK levels [2]. The studies lead us to speculate that the downregulation of miR-23b-3p may participate in muscle damage through targeting immune cells and inflammatory mediators (Figure 5).

The downregulation of serum miR-146a-5p has negatively correlated with CRP, while there was no association with MSAs, CK, and organ involvement in DM patients. This may be explained by the knowledge of miR-146a-5p involved in the inflammation progression and autoimmune diseases [31]. In line with our study, Okada et al. also found that serum miR-146a-5p levels tended to be downregulated in patients with dermatomyositis with CC genotype, which associated with disease activity [32]. Recent findings further showed that miR-146a-5p downregulated in the macrophage of DM patients contributes to the infiltration of inflammatory macrophages in DM by targeting TRAF6 and affecting the IL-17/ICAM-1 pathway [8]. The overexpression of miR-146a is induced by LPS-mediated inflammatory responses and leads to the downregulation of target genes interleukin-1 receptor-associated kinase (IRAK) 1 and tumor necrosis factor receptor-associated factor 6 (TRAF6), thus serving as a negative feedback for immune activation [33]. These studies may prompt us to speculate that downregulated serum miR-146a-5p overexpresses its target genes IRAK1 and TRAF6, then activates the NF-*κ*B signaling pathway and induces the production of type I interferons, OAS1, and Mx1, and consequently contributes to the pathogenesis of DM (Figure 5).

miR-146b-5p is a family of miR-146, sharing a seed sequence. Therefore, we further studied the serum expression of miR-146b-5p in DM patients. Our results showed that the high expression of serum miR-146-5p was negatively correlated with ESR and CRP, respectively. These results are in agreement with Eisenberg et al.'s study who found that miR-146b-5p was overexpressed in DM patients [3]. miR-146b-5p level was upregulated in rheumatoid arthritis patients compared to healthy controls. The upregulation of miR-146b-5p was shown to be related to the infiltration of IL-17-producing T cells in the RA synovium and to a higher expression of IL-17 within T cells which expanded from the peripheral blood mononuclear cells (PBMCs) [34]. In addition, we also found that serum expression of miR-146b-5p in DM patients with cancer was higher than that in patients without. Previous studies reported that expression of miR-146b-5p significantly increased in solid cancers. The upregulation of miR-146b-5p could downregulate the expression levels of its target genes to mediate the proliferation, invasion, and migration of cancer cells [35–37]. These studies indicated that miR-146b-5p may be to some extent a biomarker for cancer associated with DM. Due to the small sample size of cancer associated with DM in the study, the result should be confirmed by prospective studies in a large sample size of cancer associated with DM.

In our study, we also found that patients with anti-Jo-1, anti-PL-7, anti-EJ, and anti-MDA5 antibodies are more likely to have interstitial lung disease, while the DM patients with an anti-TIF1-*γ* antibody mainly were without ILD (Table 1), which is consistent with previous studies [12, 38]. Unexpectedly, our study did not find that the four serum immune-related miRNAs (miR-23b-3p, miR146a-5p, miR-146b-5p, and miR-150-5p) were not specifically associated with ILD in DM. However, there were interesting findings that (1) serum levels of miR-146b-5p in DM patients with ILD were lower than those in patients without ILD (*P* = 0.013) but did not reach statistical significance between DM patients with ILD and HCs (*P* > 0.05) and (2) serum expression of miR-150-5p in DM with UIP was much lower than that in DM with NSIP and OP. Therefore, further study should be conducted to explore the exact roles of miR-146b-5p and miR-150-5p in DM with different types of ILD. In accordance with our study, Honda et al. also suggested that serum miR-150 was downregulated in systemic sclerosis (SSc) but not significantly correlated with SSc with ILD [39].

There are some limitations of this explorative study: firstly, the study has cross-sectional design. Data should therefore be interpreted with some caution until future verification in large validation cohorts. Secondly, the comparatively small numbers of patients with MSAs were analyzed for miRNA expression which may lead to bias results. Another drawback is that a functional study for each miRNA was not performed. Further study is undergoing to explore the function of these serum miRNAs and their pathological role in DM.

However, to our knowledge, this is the first time to link the expression levels of specific serum miRNAs to MSA profiles in DM. Serum immune-related miRNAs may be involved in the pathogenesis and manifestations of the various DM subtypes.

## Figures and Tables

**Table 1:** Clinical features of the DM patients.

	All	DM with ILD	DM without ILD	*P* ^a^ value
Number of patients (*n*)	49	25	24	
Age at onset (mean ± SD, years)	47.26 ± 11.44	48.16 ± 9.72	46.33 ± 13.15	0.582
Sex (F/M)	33/16	19/6	14/10	
Disease duration, mean of IQR months	19.04 (3.5–24)	21.56 (2.5–45)	17.22 (4.0–22.0)	0.561
Number of initial treatment, *n* (%)	17 (34.6%)	9	8	
Serological features				
CRP (mg/dL)	0.64 ± 0.77	0.71 ± 0.67	0.54 ± 0.82	0.133
ESR (mm/h)	19.22 ± 15.73	15.90 ± 3.18	12.74 ± 2.60	0.006
CK (*μ*/L)	609 ± 1190.2	596.96 ± 1234.59	621.58 ± 242.37	0.562
LDH (*μ*/L)	315.25 ± 235.00	299.2 ± 219.59	332.69 ± 254.49	0.741
^∗∗^Ferritin (ng/mL)	535.29 ± 545.91	612.92 ± 624.26	408.26 ± 378.63	0.336
Anti-Jo-1 antibody, *n* (%)	5 (10.2%)	5	0	
Anti-MDA5 antibody, *n* (%)	11 (22.4%)	10	1	
Anti-TIF1-*γ* antibody, *n* (%)	8 (16.3%)	0	8	
Anti-NXP2 antibody, *n* (%)	6 (12.2%)	1	5	
Anti-PL-7 antibody, *n* (%)	4 (8.2%)	4	0	
Anti-PL-12 antibody, *n* (%)	0	0	0	
Anti-OJ antibody, *n* (%)	0	0	0	
Anti-EJ antibody, *n* (%)	2 (4.8%)	2	0	
Anti-SAE antibody, *n* (%)	2 (4.8%)	1	1	
Anti-Mi2 antibody, *n* (%)	2 (4.8%)	0	2	
Anti-SRP antibody, *n* (%)	1 (2.4%)	1	0	
HMGCR antibody, *n* (%)	1 (2.4%)	0	1	
MSA negative, *n* (%)	9 (18.4%)	2	7	
Pulmonary function test, *n* (%)	26 (53%)	19	7	
FEV1	80.68 ± 19.11	75.71 ± 19.03	94.71 ± 11.77	0.022
FVC	83.65 ± 21.59	79.02 ± 20.68	96.22 ± 21.15	0.071
DLCO	61.29 ± 18.29	56.27 ± 17.78	75.52 ± 11.8	0.014
PGA-VAS	6.12 ± 1.67	6.36 ± 1.38	5.87 ± 1.94	0.321

Average values or numbers of each group are shown. Standard deviation (SD), interquartile range (IQR), or percentages are shown. DM: dermatomyositis; ILD: interstitial lung disease; CRP: C-reactive protein; ESR: erythrocyte sedimentation rate; CK: creatine kinase; LDH: lactate dehydrogenase; FEV1: forced expiratory volume in one second; FVC: forced vital capacity; DLCO: carbon monoxide diffusing capacity; PGA: physician's global assessment; VAS: visual analogue scale. ^∗∗^The results of ferritin were partially missed. Data were available for 29 patients. *P* ^a^ value: between DM with ILD and DM without ILD patients.

**Table 2:** 

miRNA/snRNA	Primer sequences
miR-23a-3p	Forward: CCGATCACATTGCCAGGGATTTCC
miR-23b-3p	Forward: GCGATCACATTGCCAGGGATTACC
miR-146a-5p	Forward: GCGTGAGAACTGAATTCCATGGGTT
miR-146b-5p	Forward: CCGCTGAGAACTGAATTCCATAGGCT
miR-150-5p	Forward: TCTCCCAACCCTTGTACCAGTG
U6	Forward: GGAACGATACAGAGAAGATTAGC
	Reverse: TGGAACGCTTCACGAATTTGCG

**Table 3:** Serum/plasma expression of miRNAs correlated with clinical phenotypes in DM.

miRNAs	Regulation	Clinical phenotypes	Ref
miR-4442	Up	Skeletal disease activities	[23]
miR-146a	Down	Inflammation	[8]
Let-7b	Up		[11]
miR-3907	Up	Disease activity	
miR-4299	Down		
miR-7	Down	Gottron's eruption	[26]
miR-223	Down	Gottron's papules	[24]
miR-23b-3p	Down	CK	Our study
miR-146a-5p	Down	CRP	
miR-146b-5p	Up	ESR, CRP, and DM complicated with cancer	
miR-150-5p	Down	Anti-MDA5 and anti-NXP2 antibody	

DM: dermatomyositis; CK: creatine kinase; CRP: C-reactive protein; ESR: erythrocyte sedimentation rate; anti-MDA5: anti-melanoma differentiation-associated gene 5; anti-NXP2: anti-nuclear matrix protein-2

## Data Availability

The data used to support the findings of this study are available from the corresponding author upon request.

## Supplementary Materials

Supplementary Materials Supplementary Table 1 shows the clinical features of the DM patients associated with PBMCs.
