# Supplementary material for: Specific Autoantibodies and Clinical Phenotypes Correlate with the Aberrant Expression of Immune-Related MicroRNAs in Dermatomyositis
Source: J Immunol Res. 2019 Feb 19;2019:2927061. doi: 10.1155/2019/2927061 (PMC6399529; doi:10.1155/2019/2927061)
Supplement: Supplementary Materials — Supplementary Table 1 shows the clinical features of the DM patients associated with PBMCs. [file 2927061.f1.pdf]

Supplemenatry Table1 Clinical features of the DM patients

	ALL
Number of patients, n	23
Age at onset, mean ±SD (years)	45.65±13.94
Sex (F/M)	19/4
Disease duration, mean of IQR months	63 (5–36)
Serological features	
CRP, mg/dl	0.38±0.38
ESR, mm/h	17.82±17.22
CK, U/L	386.18±808.09
LDH, U/L	323.27±174.88
Ferritin, ng/ml	466.95±1468.87
Anti-Jo-1 antibody, n (%)	4(17.3%)
Anti-MDA5 antibody, n (%)	5(21.7%)
Anti-TIF1γ antibody, n (%)	1(4.3%)
Anti-NXP2 antibody, n(%)	6(26%)
Anti-PL-7 antibody, n(%)	1(4.3%)
Anti-PL-12 antibody, n(%)	0
Anti-OJ antibody, n(%)	0
Anti-EJ antibody, n(%)	0
Anti-SAE antibody, n(%)	0
Anti-Mi2 antibody, n(%)	1(4.3%)
Anti-SRP antibody, n(%)	0
HMGCR antibody, n(%)	0
MSA negative, n(%)	5(21.7%)
Number of ILD, n(%)	11(47.8%)

Average values or numbers of each group are shown. Standard deviation (SD), interquartile range (IQR) or percentages are shown in table. DM: dermatomyositis; CRP: C-reactive protein; ESR: erythrocyte sedimentation rate; CK: creatine kinase; LDH:lactate dehydrogenase; ILD: interstitial lung disease.
